# Changing Trends in the Etiology of Cirrhosis in Türkiye: A Multicenter Nationwide Study

**DOI:** 10.5152/tjg.2024.23572

**Published:** 2024-10-01

**Authors:** Enver Üçbilek, Abdullah Emre Yıldırım, Zeynep Ellik, İlker Turan, Büşra Haktanıyan, Berk Orucu, Mehmet Demir, Mukaddes Tozlu, Nimet Yılmaz, Yasemin Balaban, Ahmet Uyanıkoğlu, Mesut Akarsu, Ramazan Yolaçan, Orhan Sezgin, Kendal Yalçın, Murat Aladağ, Bilal Toka, Ayşe Kefeli, Aslı Örmeci, Alper Yurçi, Sami Fidan, Genco Gençdal, Ufuk Avcıoğlu, Ayhan Hilmi Çekin, Berat Ebik, Eylem Karatay, Murat Akyıldız, Aydın Şeref Köksal, Osman C. Özdoğan, Zeki Karasu, Ramazan Idilman, of the TASL Viral Hepatitis Special Interest Group Member

**Affiliations:** 1 Department of Gastroenterology, Mersin University Faculty of Medicine, Mersin, Türkiye; 2 Department of Internal Medicine, İstanbul Arel University Faculty of Medicine, İstanbul, Türkiye; 3 Department of Internal Medicine, Karaman Training and Research Hospital, Ankara, Türkiye; 4 Department of Gastroenterology, Ege University Faculty of Medicine, İzmir, Türkiye; 5 Department of Internal Medicine, Ankara University Faculty of Medicine, Ankara, Türkiye; 6 Department of Gastroenterology, Mustafa Kemal University Faculty of Medicine, Hatay, Türkiye; 7 Department of Gastroenterology, Sakarya University Faculty of Medicine, Sakarya, Türkiye; 8 Department of Gastroenterology, Sanko University Faculty of Medicine, Gaziantep, Türkiye; 9 Department of Gastroenterology, Hacettepe University Faculty of Medicine, Ankara, Türkiye; 10 Department of Gastroenterology, Harran University Faculty of Medicine, Şanlıurfa, Türkiye; 11 Department of Gastroenterology, Dokuz Eylül University Faculty of Medicine, İzmir, Türkiye; 12 Department of Gastroenterology, Dicle University Faculty of Medicine, Diyarbakır, Türkiye; 13 Department of Gastroenterology, Medipol University Faculty of Medicine, İstanbul, Türkiye; 14 Department of Internal Medicine, Karatay University Faculty of Medicine, Konya, Türkiye; 15 Department of Gastroenterology, Tokat Gaziosmanpaşa University Faculty of Medicine, Tokat, Türkiye; 16 Department of Gastroenterology, İstanbul University Istanbul Faculty of Medicine, İstanbul, Türkiye; 17 Department of Gastroenterology, Medicana Atakoy Hospital, Kayseri, Türkiye; 18 Department of Gastroenterology, Karadeniz Technical University Faculty of Medicine, Trabzon, Türkiye; 19 Department of Gastroenterology, Koç University Faculty of Medicine, İstanbul, Türkiye; 20 Department of Gastroenterology, Ondokuz Mayıs University Faculty of Medicine, Samsun, Türkiye; 21 Department of Gastroenterology, University of Health Sciences, Antalya Training and Research Hospital, Antalya, Türkiye; 22 Department of Gastroenterology, University of Health Sciences, Gazi Yaşargil Training and Research Hospital, Diyarbakır, Türkiye; 23 Department of Gastroenterology, İstinye University Faculty of Medicine, İstanbul, Türkiye; 24 Department of Gastroenterology, Ankara Etlik City Hospital, Ankara, Türkiye; 25 Department of Gastroenterology, Marmara University Faculty of Medicine, İstanbul, Türkiye; 26 Department of Gastroenterology, Ankara University Faculty of Medicine, Ankara, Türkiye

**Keywords:** Cirrhosis, etiology, viral hepatitis, metabolic dysfunction-associated steatotic liver disease, hepatocellular carcinoma

## Abstract

**Background/Aims:**

The aim of our study was to investigate the underlying causes behind the etiology of cirrhosis in Türkiye.

**Materials and Methods:**

The study was comprised of patients with cirrhosis located in the gastroenterology clinics of 28 centers in Türkiye between January 2000 and June 2021.

**Results:**

The study group consisted of 4953 cirrhotic patients (median age: 62.2 years, male / female: 58% / 42%). Among the patients, 39% of the patients were compensated, and 61% were decompensated. Furthermore, 47.5% had Child-Pugh class A, 38% had Child-Pugh class B, and 14.5% had Child-Pugh class C. The most frequent complaints were abdominal bloating (28%). Ascites (54.2%) was the most common manifestation of decompensation. The median Child-Pugh and MELD-Na scores were 7.0 and 10.0, respectively. The most common cause of cirrhosis was chronic viral hepatitis (43%), followed by cryptogenic cirrhosis (CC) (19%), metabolic dysfunction-associated steatotic liver disease (MASLD)-related cirrhosis (13%), and alcohol-related cirrhosis (11%). Among the 950 patients with CC, 416 had metabolic abnormalities. If these 416 CC patients with metabolic abnormalities were categorized as having MASLD-related cirrhosis, the proportion of MASLD-related cirrhosis increased to 21%. Thirteen percent of the patients were diagnosed with HCC, while 4% had extrahepatic malignancy. Female breast cancer (18%) and colorectal cancer (18%) were the most frequent extrahepatic malignancies.

**Conclusion:**

Viral hepatitis remains the main cause of cirrhosis in Türkiye. However, its prevalence seems to be declining, whereas the prevalence of steatotic liver disease-related cirrhosis is increasing.

**Main Points**

- In Türkiye, chronic hepatitis B infection remains the most common cause of cirrhosis.
- In the past 10 years, the prevalence of cirrhosis associated with steatotic liver disease has been increasing.
- The most frequently observed extrahepatic cancers in cirrhotic patients are female breast cancer and colorectal cancer.

## Introduction

Cirrhosis can develop as a consequence of various chronic liver diseases (CLD) and is a major public health concern, causing more than 2 million deaths each year worldwide.^1^ Common causes of CLD include hepatitis B virus (HBV), hepatitis C virus (HCV), alcohol-related liver disease (ALD), metabolic dysfunction-associated steatotic liver disease (MASLD), autoimmune liver diseases, and metabolic and vascular liver diseases.^2-6^ The etiologies of cirrhosis are related to geographical regions.^2,3^ While HCV infection and steatotic liver disease (SLD) are the most common etiologies of cirrhosis in Western countries, HBV infection stands as the predominant cause of cirrhosis and hepatocellular carcinoma (HCC) in East Asia, the Western Pacific region, Sub-Saharan Africa, and parts of the Eastern European Region.^2-4^ In recent years, the significant success of viral hepatitis elimination programs and increased access to highly effective antivirals for chronic HCV and HBV infections have probably contributed to the notable reduction in viral etiology as a cause of CLD. Obesity, diabetes epidemics, and increasing rates of alcohol abuse have also reshaped the epidemiology of CLD and cirrhosis worldwide.^3-6^

In Türkiye, viral hepatitis (HBV and HCV) is still the most frequent cause of CLD, cirrhosis, and HCC in the adult population.^7-9^ The global epidemic of obesity and the prevalence of obesity-associated diseases have also increased. The prevalence of MASLD is estimated to be above 30% in Türkiye.^10^ This prevalence is higher in patients with type 2 diabetes mellitus, patients with hyperlipidemia, and obese patients. In addition, increasing awareness of MASLD, autoimmune, and metabolic diseases, along with more widespread struggle against HCV and HBV infections, might have also caused similar shifts in the etiology of CLD in Türkiye.^11^ Data regarding the etiology of cirrhosis and the clinical and demographic characteristics of patients with cirrhosis in a large cohort of Turkish patients are limited. The aim of this study was to investigate the underlying causes behind the etiology of cirrhosis in Türkiye over the last 2 decades.

## Materials and Methods

This was a retrospective, multicenter, cross-sectional cohort study. It was conducted in gastroenterology outpatient clinics of 28 tertiary centers located in Türkiye between January 2000 and June 2021. A special electronic case report form (CRF) was designed by the Turkish Association for the Study of the Liver for data collection and recording. Each center entered the relevant data, including patients’ demographic and clinical parameters, into the CRF. Finally, the data were reviewed and analyzed by the coordinating center. International Classification of Diseases-10 (ICD-10) codes were used to identify cirrhosis, which was defined clinically, biochemically, and histologically when available. At the time of diagnosis, the signs and symptoms, clinical stages of cirrhosis, and specific complications of decompensation were recorded. Imaging findings were reviewed to assess the development of HCC and portal vein thrombosis (PVT). The study was approved by the local Ethical Committee of Marmara University School of Medicine (approval number: 09.2019.390, date: April 6,2019).

## Methods

Serum aspartate aminotransferase (AST), alanine aminotransferase (ALT), alkaline phosphatase (ALP), gamma-glutamyl transpeptidase (GGT), bilirubin, and complete blood cell counts were measured in the local laboratory of each center. Serological markers for viral infections (HBsAg, anti-HBs, HBeAg, anti-HBe, anti-HBc IgG, anti-HCV, anti-HAV IgM, anti-HEV, anti-cytomegalovirus, anti-Epstein-Barr virus, and anti-herpes simplex virus), serum iron, ferritin, ceruloplasmin, and alpha-1 antitrypsin levels were measured. Serological studies were performed for anti-nuclear, anti-liver kidney membrane-1, anti-smooth muscle, and antimitochondrial antibodies. To confirm the diagnosis of cirrhosis, its complications, or HCC, abdominal ultrasonography was performed on all patients.

### Definitions

The etiology of cirrhosis was categorized as HBV infection, HCV infection, hepatitis delta infection (HDV), MASLD, ALD, autoimmune liver diseases (autoimmune hepatitis (AIH), primary biliary cholangitis (PBC), primary sclerosing cholangitis (PSC)), metabolic liver disease (hemochromatosis, Wilson’s disease, alpha-1 antitrypsin deficiency), and vascular liver disease (Budd-Chiari syndrome) using ICD-10 diagnostic codes. Etiologies were diagnosed based on international criteria. Child-Pugh, MELD, and MELD-Na scores were used to assess the severity of cirrhosis.^12-14^ Laboratory parameters, imaging findings, and the clinical course of the disease were reviewed. Decompensations of cirrhosis, including ascites, variceal bleeding (VB), spontaneous bacterial peritonitis, hepatic encephalopathy (HE), hepatorenal syndrome (HRS), hepatopulmonary syndrome (HPS), portopulmonary hypertension, PVT, and HCC were evaluated. The development of extrahepatic malignancies was determined.

The primary outcome of this study was to investigate the change in the etiology of cirrhosis during the last 2 decades. The secondary outcome was to determine the clinical and demographic characteristics of the observed cirrhotic patients.

### Statistical Analysis

The chi-squared test was used to investigate relationships between categorical variables. Bonferroni corrections were applied for pairwise comparisons after significant chi-squared test results were found. Mean ± SD, 95% CIs, and median and interquartile ranges were used to describe the numerical data. Frequencies and percentages were used for categorical data. Statistical analysis was performed with the Statistical Package for the Social Sciences version 24.0 (IBM SPSS Corp.; Armonk, NY, USA). A *P*-value < .05 was accepted as statistically significant.

## Results

A total of 5018 cirrhotic patients were included in the study. Sixty-five patients were excluded from the study due to lack of data. All patients were Caucasian. The median age was 62.2 years (range: 18-90 years), and most were males (57.8%). The median body mass index was 27.0 kg/m^2^ (range: 15.6-57.1 kg/m^2^). Upon admission, 39.4% of the patients classified as compensated, while 60.6% were decompensated. Among them, 47.5% had Child-Pugh class A, 38.0% had Child-Pugh class B, and 14.5% had Child-Pugh class C. The most frequent complaints of cirrhotic patients were abdominal bloating (28.1%), liver test abnormalities on routine laboratory examination (26%), variceal hemorrhage (11.7%), jaundice (4.2%), unconsciousness (3.7%), and pruritus (2.8%). Ascites (54.2%) were the most common manifestation of decompensation, followed by VB (29.2%), HE (15.9%), HRS (1.7%), and HPS (1.1%). Median Child-Pugh and MELD-Na scores were 7.0 (range: 5-9) and 10.0 (range: 8-15), respectively. The baseline characteristics of the patients are provided in Table 1.

The most common cause of cirrhosis was chronic viral hepatitis (43%). Chronic hepatitis B was the main etiology of cirrhosis in the overall cohort (30.7%), followed by cryptogenic cirrhosis (CC) (19.2%), chronic hepatitis C (12.3%), MASLD-related cirrhosis (12.6%), ALD (10.5%), autoimmune liver diseases (7%; AIH in 3.7%, PBC in 2.7%, and PSC in 0.6%), delta-cirrhosis (3.2%), Wilson’s disease (2.1%), Budd-Chiari syndrome (1.4%), and miscellaneous causes (1%). Notably, among the 950 patients with CC, 416 (43.7%) had metabolic abnormalities, including diabetes mellitus, hypertension (HT), and/or metabolic syndrome (MetS). When these 416 CC patients with metabolic abnormalities were categorized as having MASLD-related cirrhosis, the proportion of MASLD-related cirrhosis increased from 12.6% (n = 626) to 20.8% (n = 1042), while CC decreased from 19.2% (n = 950) to 10.7% (n = 534).

When the etiologies of cirrhosis diagnosed before and after 2010 were compared, the proportion of MASLD-related cirrhosis significantly increased after 2010 (from 9% to 24%, *P* < .05; respectively). On the other hand, the proportion of viral hepatitis-related etiology, such as HBV (from 41% to 27%, *P* < .05) and HDV (from 8% to 2%, *P* < .05) decreased after 2010 (Table 2).

The etiology of cirrhosis varies across different geographical regions of Türkiye. Hepatitis B virus-related cirrhosis is more frequently seen in the Southeast and Central Anatolia, ALD in the Aegean and Mediterranean regions, and HCV in the Mediterranean and Southeast Anatolia regions, while MASLD-related cirrhosis is more commonly seen in the Marmara and Aegean regions (Table 3).

The reasons for each patient’s admission to the hospital varied according to the etiology of cirrhosis at diagnosis. The most common admission symptom was ascites in patients with ALD (42%), while liver test abnormalities during routine laboratory evaluation were found in patients with viral etiology-related cirrhosis (31.5%). Patients with MASLD-related cirrhosis were mostly diagnosed incidentally or after the evaluation of elevated liver test levels (29.9%).

On admission, 538 patients (12.7%) had HCC, while 141 patients (3.8%) had an extrahepatic malignancies. Female breast cancer (18%), colorectal cancer (18%), and hematological malignancies (14%) were the most common extrahepatic malignancies. Patients with HCC were significantly older (64.9 ± 11.6 years vs. 60.2 ± 13.9 years, *P* < .001), and were predominantly male (75.9% vs. 24.1%, *P* < .001). They had a higher prevalence of viral etiology (65.6% vs. 58.6%, *P* < .001) compared to those without HCC (Table 4). Most HCC patients had HBV-associated HCC (47.2%), followed by HCV-associated HCC (15.2%) and HDV-associated HCC (3.1%). Metabolic dysfunction-associated steatotic liver disease-associated HCC was the most common non-viral etiology (11.3%).

## Discussion

The present nationwide multicenter study investigated changing trends in the etiology of cirrhosis in Türkiye over the past 2 decades. Viral hepatitis remains the most common cause of cirrhosis, but it has a significantly declining prevalence. On the other hand, the prevalence of MASLD- and ALD-related cirrhosis is significantly increasing. In addition, the geographical region affected the etiology of cirrhosis. Hepatitis B virus-related cirrhosis is more frequently seen in Southeast and Central Anatolia, while MASLD-related cirrhosis is more frequently seen in the Marmara and Aegean regions. A nationwide epidemiological study in Türkiye, the TURHEP study, revealed an HBsAg positivity of about 4% and an anti-HBcIgG positivity of about 31% among adults in 2009. It was estimated that more than 2 million adults in Türkiye were HBsAg-positive. Unfortunately, only 12% were aware of their condition.^8^ Hepatitis B virus vaccination is the most effective way to prevent HBV infection. Since 1998, 3 HBV vaccine doses have been added to the childhood vaccination program in Türkiye. Thus, the incidence of acute HBV infection has gradually decreased in children, based on the Health Ministry data.^15^ Increased awareness regarding elimination programs and the introduction of effective antivirals may have contributed to the decrease in the prevalence of the viral etiology found in the study. We believe that the prevalence of viral-etiology-related cirrhosis in Türkiye will decrease even more in the future.

The etiologies of CLD and cirrhosis continue to change over time worldwide. The prevalence of MASLD has increased gradually on a global scale over the past 2 decades.^16^ The present study found an increase in MASLD-related cirrhosis. The proportions of CC and MASLD-related cirrhosis were 19% and 13%, respectively. However, when we assume that some patients with CC originated from MASLD, the proportion of MASLD-related cirrhosis increases from 13% to 21%. This result confirms previous studies demonstrating that MASLD was the most common cause of CLD and cirrhosis.^5,6^ In addition, these results indicate that MASLD is emerging as a common cause of CLD and cirrhosis in Türkiye and suggest that patients with cirrhosis should be carefully evaluated for metabolic abnormalities before establishing a diagnosis of CC. By comparing the etiology of cirrhosis and the characteristics of the cirrhotic patients diagnosed before and after January 2010, this study finds that the prevalence of viral hepatitis-related cirrhosis is declining while the prevalence of MASLD-related cirrhosis is increasing. These changing trends in the etiology of cirrhosis are important for planning and developing effective prevention and treatment strategies. The current study highlights the importance of implementing effective interventions to reduce the burden of risk factors for MASLD in this respect.

Hepatocellular carcinoma is one of the most common cancers in the world. Regardless of the etiology, the annual incidence of HCC in cirrhotic patients is between 1% and 4%.^17^ The risk factors for HCC development are the presence of cirrhosis, active hepatic inflammation, viral etiology, male gender, and older age. In this study, 13% of the cirrhotic patients were diagnosed with HCC during admission. The patients with HCC were older and were predominantly male. Most of the HCC patients had viral hepatitis-associated HCC, and MASLD-associated HCC was the second most prevalent type of HCC in the observed patients. These results confirm the findings of previous studies.^5,11,18-20^ In addition, 4% of the cirrhotic patients had extrahepatic malignancy. The most common malignancies were female breast cancer, colorectal cancer, and hematological malignancies. Limited data are available in the literature regarding extrahepatic cancers in patients with cirrhosis.^19^ Allaire et al found that the rate of extrahepatic malignancies was 5.3%, and the most common cancer types in these malignancies were lymphoid tissue-related, oral cavity, colon, and lung cancers.^19^

The current study was a large, multicenter collaborative study, but it had several limitations. First, it utilized a retrospective design and had missing data. Secondly, the study group included cirrhotic patients who were admitted and followed up in liver disease outpatient clinics of tertiary referral centers throughout Türkiye. Therefore, the results may not represent the entire Turkish population.

In conclusion, viral hepatitis is still the main cause of cirrhosis in Türkiye. However, the prevalence of viral hepatitis-related cirrhosis is beginning to decline, while the prevalence of MASLD-related cirrhosis has increased over the last 2 decades.

## Figures and Tables

**Table 1.** Characteristics of all Cirrhotic Patients

Characteristics	n = 4953
Age (years)	62.2 (18-90)
Gender (F/M)	42%/58%
BMI (kg/m^2^)	27.0 (15.6-57.1)
Glucose (mg/dL)	106 (91-144)
ALT (U/L)	28 (19-48)
AST (U/L)	39 (27-61)
GGT (U/L)	57 (31-107)
ALP (U/L)	109 (80-154)
Albumin (mg/dL)	3.5 (2.9-4.0)
Total bilirubin (mg/dL)	1.3 (0.8-2.1)
Creatinine (mg/dL)	0.79 (0.63-0.97)
INR	1.22 (1.1-1.4)
MELD score	9 (7-13)
MELD-Na score	10 (8-15)
Child-Pugh score	7 (5-9)
Platelet count (/mm^3^)	107 × 10^3^ (73 × 10^3^-158 × 10^3^)

Data are given as median values (interquartile range Q1-Q3).

**Table 2.** Etiologies of Cirrhosis Before and After 2010

Etiology of Cirrhosis	Years: 2000-2009 (%)	Years: 2010-2020 (%)	*P*
HBV	41	27	<.05
HCV	12	11	.56
DELTA	8	2	<.05
MASLD	12	25	<.05
Cryptogenic	9	12	<.05
Alcohol	5	10	<.05
Autoimmune liver disease	7	7	.66
Others	8	7	.53

**Table 3.** Etiology of Cirrhosis with Respect to Geographical Regions

Etiology	Türkiye	Central Anatolia	Aegean	Marmara	Black Sea	South East	East Anatolia	Mediterranean	*P*
	%	%	%	%	%	%	%	%	
Alcohol	10.5	8.4	19.5	8.6	12.8	3.6	4.3	14.9	<.001
HBV	30.7	33.6	22.7	29.2	23.1	37.9	23.9	28.2	<.001
HCV	12.3	13.5	7.6	8.2	5.1	14.5	6.8	20.0	<.001
Cryptogenic	19.2	16.9	13.9	16.3	30.8	24.6	45.3	18.9	.001
NAFLD	12.6	9.1	17.4	25.4	16.2	8.6	5.1	9.6	.001
AIH	3.7	6.7	2.9	3.2	0.9	1.9	2.6	1.9	.001

**Table 4.** Comparison of Demographic Features, Laboratory Parameters, and Etiology in of Patients With and Without HCC

	Patients with HCC (n = 538)	Patients without HCC (n = 4415)	*P*
Age (years)	64.9 ± 11.6	60.2 ± 13.9	<001
Gender (F/M)	24.1%/75.9%	44.3%/55.7%	<.001
Baseline ALT (U/L)	51.3 ± 67.5	44.1 ± 95.8	.1
Baseline AST (U/L)	75.1 ± 109.8	53.4 ± 80.5	<.001
Baseline GGT (U/L)	125.9 ± 173.9	93.9 ± 134.9	<.001
Baseline total bilurubin (mg/dL)	2.2 ± 3.3	1.9 ± 2.5	.046
Baseline albumin (g/dL)	3.4 ± 0.7	3.5 ± 0.7	.002
Baseline INR	1.3 ± 0.37	1.3 ± 0.37	.666
Baseline MELD	10.2 ± 6.5	10.3 ± 7.2	.883
Baseline MELD-Na	12.3 ± 6.2	11.9 ± 5.7	.186
Baseline Child-Pugh score	7.4 ± 2.1	7.0 ± 2.1	.001
Etiology viral/non-viral	65.6%/35.4%	58.6%/41.4	<.001

Data are given as mean ± SD.
